# Supplementary figures and images for: The mitophagy receptor NIX induces vIRF-1 oligomerization and interaction with GABARAPL1 for the promotion of HHV-8 reactivation-induced mitophagy
Source: PLoS Pathog. 2023 Jul 17;19(7):e1011548. doi: 10.1371/journal.ppat.1011548 (PMC10374065; doi:10.1371/journal.ppat.1011548)

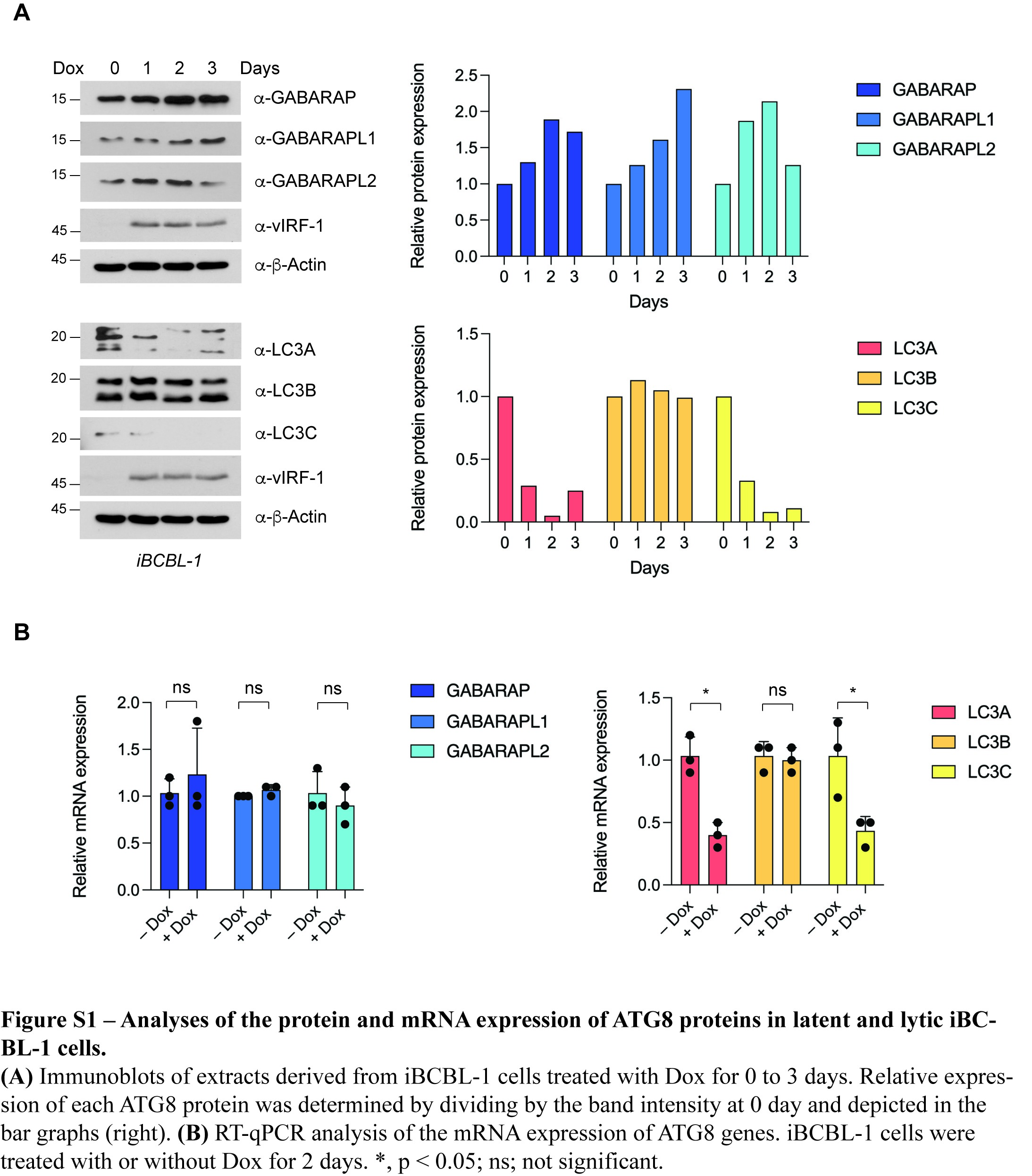

Supplement: S1 Fig — (A) Immunoblots of extracts derived from iBCBL-1 cells treated with Dox for 0 to 3 days. Relative expression of each ATG8 protein was determined by dividing by the band intensity at 0 day and depicted in the bar graphs (right). (B) RT-qPCR analysis of the mRNA expression of ATG8 genes. iBCBL-1 cells were treated with or without Dox for 2 days. *, p < 0.05; ns, not significant. (TIF) [file ppat.1011548.s001.tif]

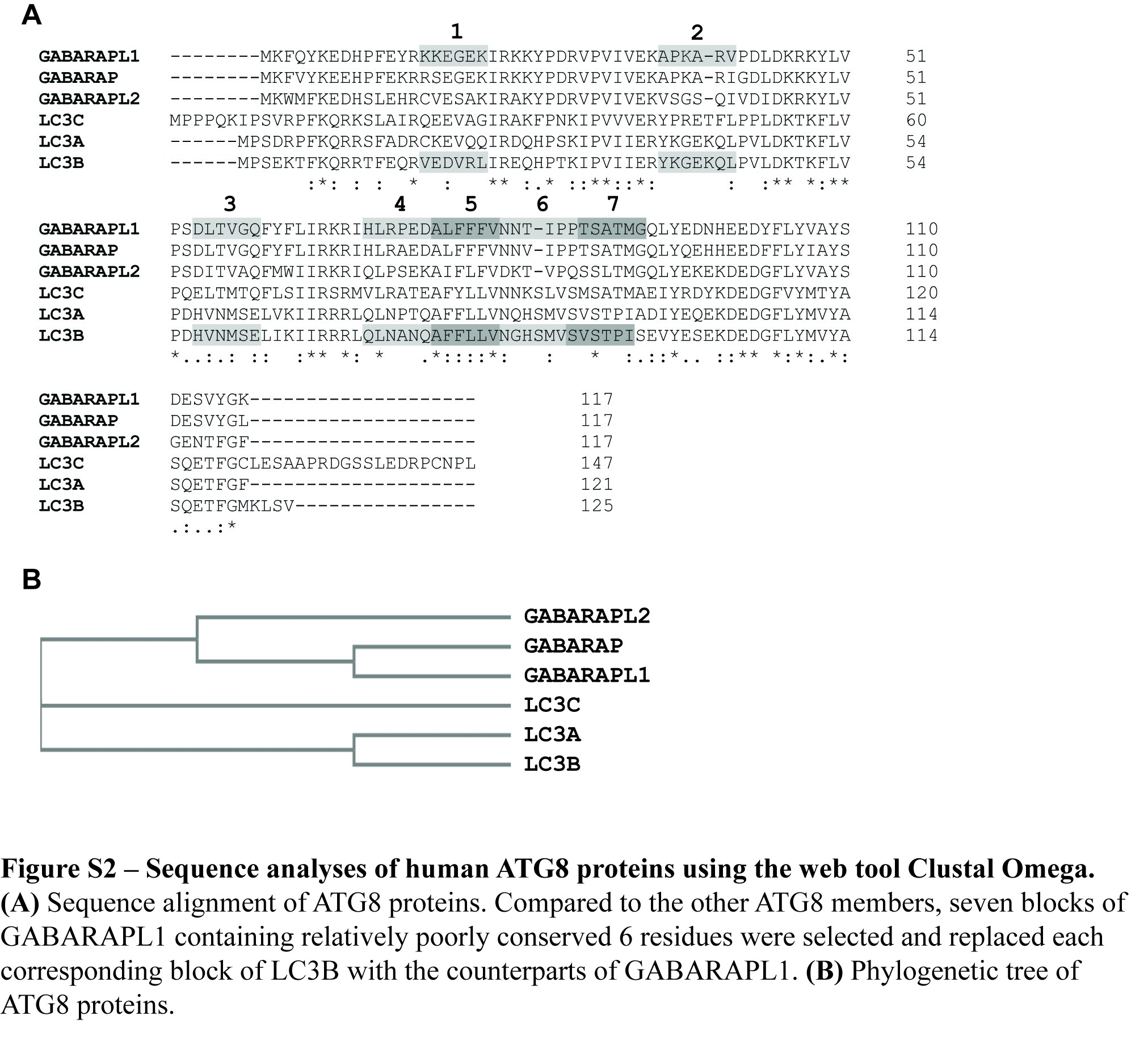

Supplement: S2 Fig — (A) Sequence alignment of ATG8 proteins. Compared to the other ATG8 members, seven blocks of GABARAPL1 containing relatively poorly conserved 6 residues were selected and replaced each corresponding block of LC3B with the counterparts of GABARAPL1. (B) Phylogenetic tree of ATG8 proteins. (TIF) [file ppat.1011548.s002.tif]

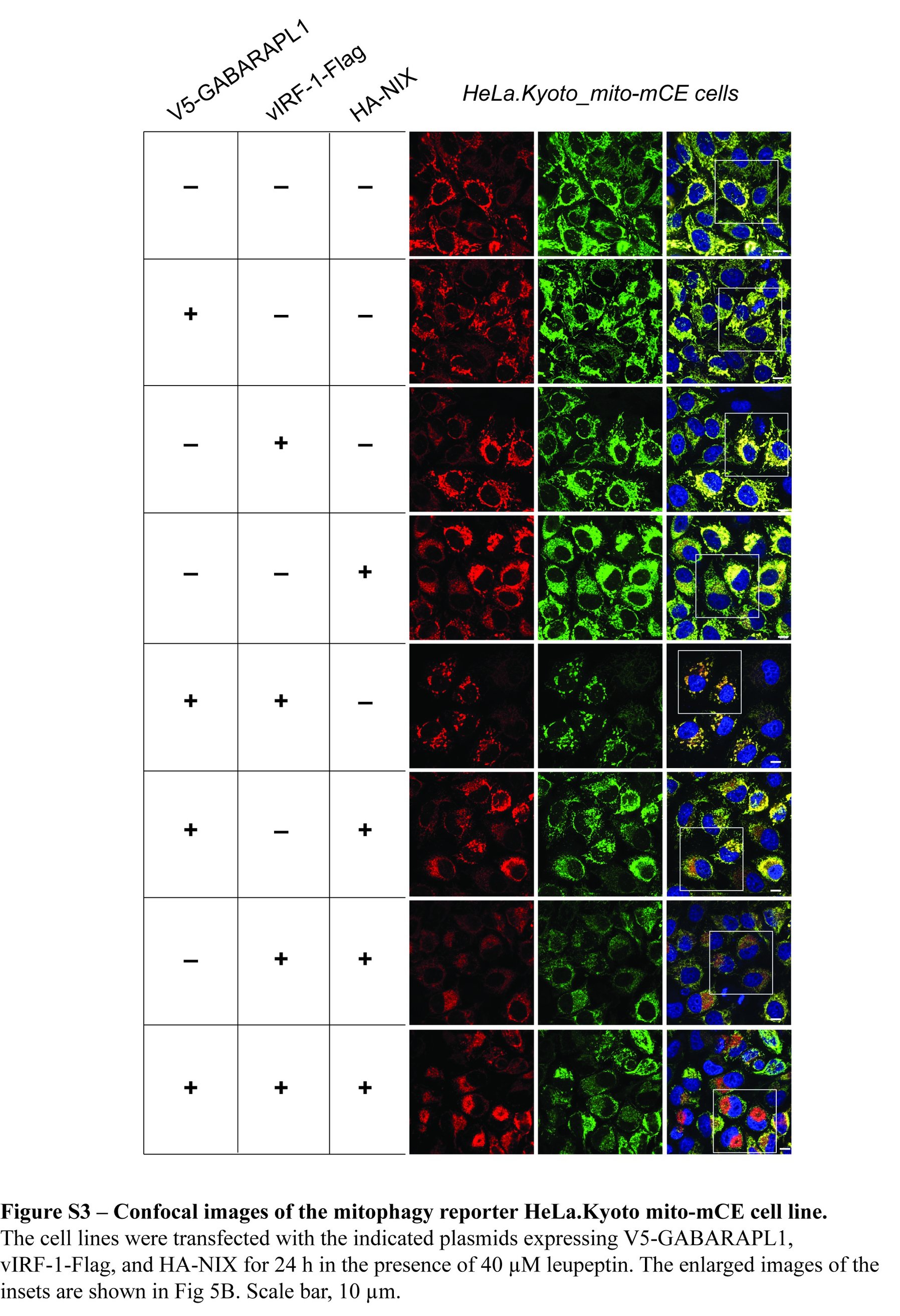

Supplement: S3 Fig — The cell lines were transfected with the indicated plasmids expressing V5-GABARAPL1, vIRF-1-Flag, and HA-NIX for 24 h in the presence of 40 μM leupeptin. The enlarged images of the insets are shown in Fig 5B. Scale bar, 10 μm. (TIF) [file ppat.1011548.s003.tif]

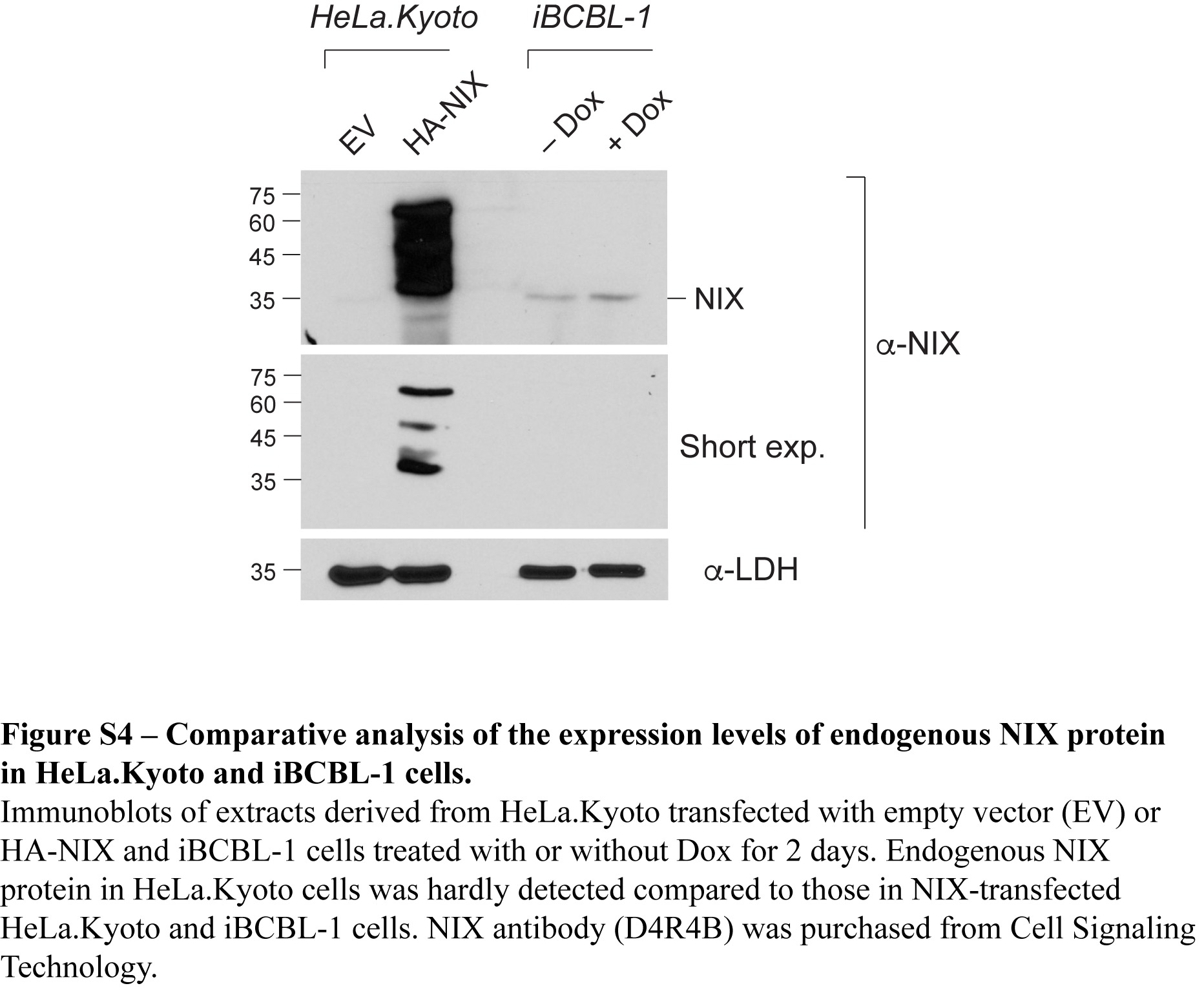

Supplement: S4 Fig — Immunoblots of extracts derived from HeLa.Kyoto transfected with empty vector (EV) or HA-NIX and iBCBL-1 cells treated with or without Dox for 2 days. Endogenous NIX protein in HeLa.Kyoto cells was hardly detected compared to those in NIX-transfected HeLa.Kyoto and iBCBL-1 cells. NIX antibody (D4R4B) was purchased from Cell Signaling Technology. (TIF) [file ppat.1011548.s004.tif]

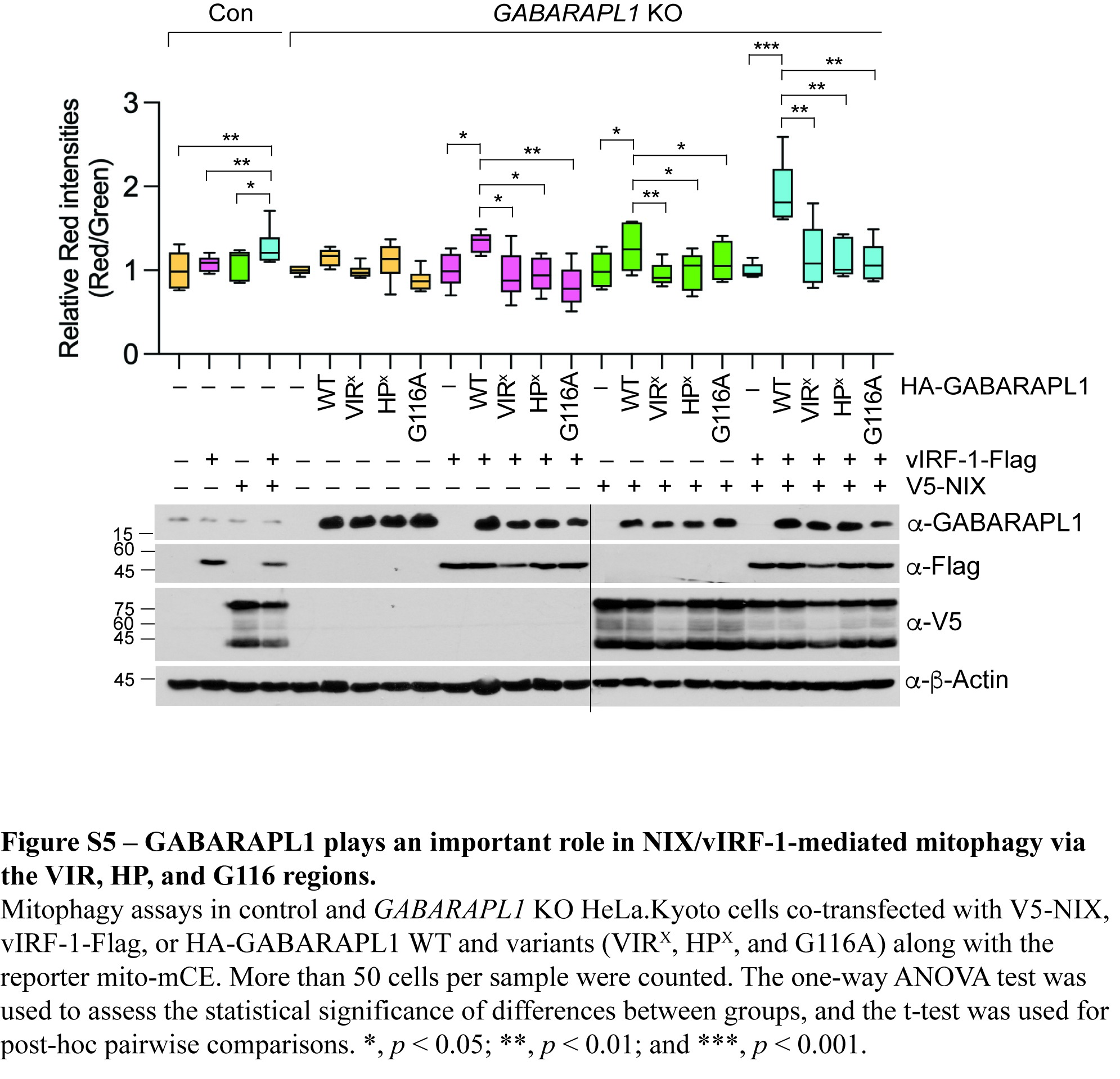

Supplement: S5 Fig — Mitophagy assays in control and GABARAPL1 KO HeLa.Kyoto cells co-transfected with V5-NIX, vIRF-1-Flag, or HA-GABARAPL1 WT and variants (VIRX, HPX, and G116A) along with the reporter mito-mCE. More than 50 cells per sample were counted. The one-way ANOVA test was used to assess the statistical significance of differences between groups, and the t-test was used for post hoc pairwise comparisons. *, p < 0.05; ** p < 0.01; and ***, p < 0.001. (TIF) [file ppat.1011548.s005.tif]
