# Supplementary material for: The mitophagy receptor NIX induces vIRF-1 oligomerization and interaction with GABARAPL1 for the promotion of HHV-8 reactivation-induced mitophagy
Source: PLoS Pathog. 2023 Jul 17;19(7):e1011548. doi: 10.1371/journal.ppat.1011548 (PMC10374065; doi:10.1371/journal.ppat.1011548)
Supplement: Attachment — Submitted filename: Response to reviewers comments.docx [file ppat.1011548.s007.docx]

Response to reviewers’ comments

We appreciate the reviewers for their constructive and insightful comments. As a result, we believe the manuscript has been improved by including new data and revising the text. Our point-by-point responses to the reviewers' comments are highlighted in blue below.

**Reviewer #1**:

In their previous work, the Choi group showed that the KSHV viral interferon regulatory factor 1 (vIRF1) binds to the autophagy receptor NIX on the outer membrane of the mitochondria to induce mitophagy and support lytic replication. In this manuscript, they explore the mechanism behind vIRF1-NIX-mediated mitophagy. They show that vIRF1 binds to the autophagy-related gene 8 (ATG8) family of proteins (central modifiers for autophagy) using recombinant proteins, with a strong preference for the GABARAPL1 in KSHV-infected cells. Through biochemical assays, they systematically mapped this interaction to 10 residues on vIRF1 and 3 residues on GABARAPL1. This vIRF1-GABARAPL1 interaction increased mitophagy in HeLa cells and further enhanced in the presence of NIX. Furthermore, NIX induces the oligomerization and stabilization of vIRF1 on the mitochondrial surface. Together, they propose a model where, during lytic reactivation, NIX is activated and oligomerizes vIRF1 on the mitochondrial surface. Oligomerized NIX and vIRF1 bind to ATG8 proteins (e.g, GABARAPL1) for mitophagy. The manuscript is well-written, and experiments are logical and well-controlled.

However, a couple of major points are unclear:

1. Is GABARAPL1 also required for vIRF1 oligomerization similar to NIX in the context of infection/iBCBL-1? Do you also see oligomerization of GABARAPL1?

- We investigated vIRF-1 oligomerization in control and *GABARAPL1* knockout (KO) iBCBL-1 cell lines, which were generated using CRISPR/Cas9. In sharp contrast to NIX, the loss of GABARAPL1 led to a robust increase in reactivation-induced vIRF-1 oligomerization (see Fig 8E). These results might be attributed to an accumulation of vIRF-1-containing mitochondria induced by mitophagy inhibition in GABARAPL1-deficient iBCBL-1 cells.
- We could not see reactivation-induced oligomerization of GABARAPL1 in virus-infected cells, including iSLK.BAC16 (see Fig 5E) and iBCBL-1 (see next figure) cells. To identify GABARAPL1 bands in immunoblotting, we included *GABARAPL1* KO cell lines.

2. Although the authors have shown that GABARAPL1 significantly increases mitophagy induced by vIRF1 and NIX (Fig. 5) in HeLa cells, this needs to be demonstrated in the context of infection/iBCBL-1. Is GABARAPL1 and its interaction with vIRF1 important for lytic reactivation? More specifically, is the interaction between vIRF1 and GABARAPL1 crucial for mitophagy during reactivation? Does this interaction have an impact on viral progeny? It might be useful to express the GABARAPL1 mutants in iBCBL-1 (in the context of endogenous GABARAPL1 KO) to see if they can rescue the phenotypes?

- As suggested, we generated *GABARAPL1* KO iSLK.BAC16 and examined mitochondria content and virus production after lytic reactivation. The results showed that productive virus replication was significantly decreased by GABARAPL1 deficiency but rescued by reconstitution with sgRNA-refractory GABARAPL1 but not the mutant VIR^X^ (see Fig 5F). Moreover, immunoblotting showed that mitochondria content (mitochondrially encoded NADH dehydrogenase subunit 1, MT-ND1) was significantly reduced by reconstitution with native GABARAPL1, but not the mutant VIR^X^, only in reactivated control and *GABARAPL1* KO iSLK.BAC16 cells (see Fig 5F), indicative of the demand of vIRF-1 interaction with GABARAPL1 for autophagic clearance of mitochondria and lytic replication.
- In addition, we generated an HHV-8 bacterial artificial chromosome 16 variant (BAC16.vIRF-1ΔGIR) encoding vIRF-1 lacking the GIR sequences (amino acids 227-236) using l-Red recombination techniques (see Fig 5A and 5B). We could observe that reactivation-induced decreases in mitochondria content (MT-ND1) and productive virus replication were inhibited in iSKL.BAC16.vIRF-1ΔGIR cells compared to iSLK.BAC16 cells (see Fig 5C and 5D).
- Our genetic studies strongly suggest that vIRF-1 interaction with GABARAPL1 is crucial in reactivation-induced mitophagy and productive replication.

Minor points that can be addressed to improve the manuscript:

1. Lines 160-164: A short explanation of the NanoBiT assay would be helpful in the main text.

- We have added the following sentences to the text.
- “In addition, to further confirm the result, we performed a luciferase-based protein fragment complementation assay, NanoLuc® Binary Technology (NanoBiT, Promega). NanoBiT is a two-subunit system, which is based on NanoLuciferase: Large BiT (LgB; 17.6 kDa) and Small BiT (SmB; 1.2 kDa) subunits fused to proteins of interest (24). When the two proteins interact, the subunits come together to form an active enzyme and generate a bright luminescent signal in the presence of substrate.”

2. Fig. 5D needs to be explained more in the main text. It was barely mentioned in lines 249-253.

- We have added the following sentences to the text. Note that Fig 5D moved to Fig 6D.
- “To confirm these results, we examined changes in mitochondria content in the above cell culture conditions, but without leupeptin, by immunoblotting MT-CO2 (*mt*DNA-encoded *c*ytochrome c *o*xidase *II*). As expected, the results showed that GABARAPL1 could promote a decrease in mitochondria content when co-transfected with either NIX or vIRF-1 and, to a greater extent, with both NIX and vIRF-1 (Fig 6D). However, the addition of leupeptin to the cell cultures blocked the decreases in mitochondria content (Fig 6D).”

3. Fig. 7C: IC50 is not an appropriate calculation to measure protein half-life (this is not used for time measurements as a variable). A decay/half-life function should be used.

- We have revised the figure (currently Fig 9C) as commented.

4. For majority of the statistical tests (Figs. 2G, 5A, 5F, 5I, 7A), ANOVA should be used and not t test since there are >2 groups. Appropriate posthoc tests can be done to calculate pairwise adjusted p values.

- As commented, we performed the statistical tests and have noted them in the figure legends.

5. The authors may want to consider placing Fig. S4 as a main figure.

- Fig S4 moved to the main figure Fig 8C and 8D. Note that we repeated the IFA experiments of Fig 8C.

**Reviewer #2**:

The manuscript by Choi and colleagues investigates the mechanism of vIRF1-induced mitophagy during KSHV lytic infection. The authors describe an interaction between vIRF1 and the autophagy related protein, GABARAPL1, and identify key residues involved in the interaction. The mitophagy receptor NIX was found to facilitate the interaction. a vIRF1 variant defective in GABARAPL1 binding had reduced ability to promote vIRF1-NIX-induced mitophagy. Lastly, a NIX CRISPR KO was found to exhibit reduced KSHV reactivation. Overall, the study provides an in depth characterization between the interaction between vIRF1 and GABARAPL1, with interesting hints to biological significance.

1. As presented, interpretation of the data presents challenges due to a lack of consistency. For example, interactions between LC3B and vIRF1 are observable in roughly half of the figures. While this may be due to the intensity of the blots, this needs to be corrected to present a consistent line of data for interpretation.

- As commented, we have repeated the *in vitro* binding experiments with more stringent washing conditions. New data are presented in Fig 1B and 3A.

2. The experimental setup of Figure 3 is unclear and would benefit from additional text. For example, why was LC3B chosen for this pairwise alignment? How well are these two proteins conserved? LC3A also does not interact well with vIRF1 in vitro--would doing this analysis with LC3A produce a similar set of regions?

- We have added the following sentences to the text. Note that we have added the sequence alignment analysis in Fig S2.
- “From the multiple sequence alignment of human ATG8 proteins using the web-based software Clustal Omega (Fig S2A), we defined seven blocks containing relatively poorly conserved 6 residues among ATG8 proteins and substituted each block in LC3B, which barely binds to vIRF-1 as shown in the previous protein interaction assays and is the most distal from GABARAPL1 in the phylogenetic tree (Fig S2B), with the counterparts of GABARAPL1 (Fig 3A).”

3. Further investigation of how a lack of vIRF1 binding to GABARAPL1 would also be useful. For instance, through the construction of the vIRF1 227-236 deletion BAC and its characterization. Or complementing the vIRF1 KO with the mutant.

- As suggested, we generated the HHV-8 BAC16 variant (BAC16.vIRF-1ΔGIR) encoding vIRF-1 lacking the GIR sequences (amino acids 227-236) using l-Red recombination techniques (see Fig 5A and 5B). We could observe that a reactivation-induced decrease in mitochondria content (MT-ND1) and productive virus replication were inhibited in iSKL.BAC16.vIRF-1ΔGIR cells compared to iSLK.BAC16 cells (see Fig 5C and 5D).

4. While the vIRF1 aggregation is interesting, its biological importance is unclear. Comments describing it as an essential step in mitophagy activation are unwarranted.

- We agree with the reviewer’s comment. We removed the words from the Abstract and revised the text.

5. The phenotype for NIX KO is minor—along this line, mitolysosomes only appear to form in a few cells—13 out of 257. Interpretation of this is confounded by the fact that lytic reactivation of iBCBL1 cells is not efficient, traditionally reported to occur for roughly half of cells in the population. Are the mitolysomes reported in Fig S4 in lytic cells? Staining for vIRF1 or another lytic marker would be sufficient.

- On average, we found about 20% of vIRF-1-positive cells from reactivated iBCBL-1 cells. We decided to perform triple-color IFA assays to detect mitolysosomes in vIRF-1-expressing iBCBL-1 cells. New data is presented in Fig. 8C.

**Reviewer #3**:

In this study, Vo et al. analyzes the molecular mechanism of how vIRF1 promotes mitophagy during the lytic cycle of KSHV, which is important for efficient virus production. This study is based on the previous findings of the Choi lab. Here, they demonstrate that vIRF1 interacts with the autophagy protein GABARAPL1, which interaction in association with the mitophagy receptor NIX is required for vIRF1-mediated mitophagy. Overall, this is very interesting study, which provides new information about the regulation of mitophagy by a KSHV protein. While most of the experiments were well executed, in some cases I found that the interpretation of some results can be improved and adding a few more data to some experiments would be helpful for better understanding the results.

Comments:

1. Line 124-125. Instead of “lytically HHV-8 infected”, I suggest using TRExBCBL-1-RTA cells undergoing lytic reactivation or during lytic reactivation using XY cells…Because lytic reactivation was induced and not lytic KSHV infection of the cells.

- As suggested, we have revised it.
- “We next examined *in cellulo* interactions of vIRF-1 with endogenous ATG8 proteins during lytic reactivation in TRExBCBL-1-RTA (simply termed iBCBL-1) cells”

2. GSTPD in Fig 1B shows direct interaction of vIRF1 with LC3C, GABARAP, GABARAPL1 and GABARAPL2 but only GABARAPL1 interacts with vIRF1 in cells. How can they explain it? Maybe instead of using whole cell lysate, cytoplasmic extract should be used for IP.

- We appreciate this comment. We repeated the experiments in Fig 1B with more stringent washing conditions and found that LC3C, GABARAP, and GABARAPL1, but not GABARAPL2, bind to vIRF-1. As suggested, we have repeated the co-IP experiment in Fig 3C with the nuclear and cytoplasmic fractions derived from reactivated iBCBL-1 cells. We could detect GABARAP and GABARAPL1 in the vIRF-1-IP complex of the cytoplasmic fraction. However, we were not able to see LC3C yet.
- Accordingly, we have added the following sentences to the text.
- “ATG8 proteins are found mainly in the cytoplasm, while vIRF-1 is found in both the nucleus and the cytoplasm. To enhance the chance of detecting ATG8 proteins binding to vIRF-1, we used the cytoplasmic fraction of reactivated iBCBL-1 cells for vIRF-1-IP. The nuclear fraction was used as a control. The results showed that only GABARAPL1 and, to a lesser extent, GABARAP were detected in the vIRF-1-IP complex precipitated from the cytoplasm (Fig 1C). Interestingly, the expression levels of GABARAPL and GABARAPL1 steadily increased after lytic reactivation in iBCBL-1 cells (Fig S1), suggesting that the GABARAP subfamily proteins may play an essential role in HHV-8 lytic infection. However, LC3C was not detected in the vIRF-1-IP complex (Fig. 1C). This result may be due to a relatively low level of LC3C in reactivated iBCBL-1 cells (Fig. S1).”

3. Fig 1D. Quality of IFA is low and lytic reactivation seems to be inefficient. 2 dpi should show much more vIRF1 expression, it is barely detectable in cells, and it is exclusively cytoplasmic. Should there not be nuclear vIRF1staining as well? Like in their previous Nature Communication paper.

- We have repeated the IFA experiment with our new rat anti-vIRF-1 antibody for better triple-color staining. We present new images of vIRF-1, GABARAPL1, and TOM20 staining of reactivated iBCBL-1 cells pre- and post-permeabilized with saponin and Triton X-100, respectively.
- Pre-permeabilization method with saponin was used to facilitate the detection of cytoplasmic and membrane-bound vIRF-1. Nuclear vIRF-1 is not detected by the method as the nuclear membrane is not permeabilized by such a mild detergent, as we demonstrated previously (Choi C.Y. et al., *Cell Death & Differentiation*, (2022) 29:451-464).

4. Fig 1F. Does the vIRF1 mutant interact with GABARAPL1 in cells? IP using cell lysate seems to be more stringent assay to test interaction than GSTPD based on Fig 1B and C. Either using transfected 293T cells or BCBL1 expressing vIRF1 WT or mutant (by lenti).

- As suggested, we performed a co-IP experiment using 293T cells transfected with vIRF-1 and the variant A255/A258 along with V5-LC3B or V5-GABARAPL1. The result showed the vIRF-1 variant could bind to GABARAPL1. (see Fig 1H).

5. Line 140-141. Lack of interaction is a confirmation of the predicated motif not being involved in ATG8 protein interaction? If G and R are not tolerated in LIR motif, how DWGRL was predicted as LIR motif in the first place?

- We performed the prediction and protein binding studies before finding the article suggesting that glycine and arginine between the core residues of LIR are not tolerated for interaction with ATG8 proteins. In addition, the web-based LIR prediction tool iLIR seems not to reflect the result of the article, as DWGRL is still predicted as a potential LIR motif with a high PSSM score of 14.

6. Fig 2B. Why DBD-IAD does not interact with GABARAPL1 if DBD of vIRF1 is required for their interaction? What is the band in the first lane of the input?

- We replace it with an image that is longer exposed, in which GABARAPL1 could be precipitated with the DBD-IAD.
- Sorry for the confusion. We missed loading the input on the gel in the pull-down assay. The band in the first lane of the bottom panel is GST used in the pull-down.

7. Line 171. Subtitle is missing for the description of Fig 3, which shows the mapping of interaction domain in GABARAPL1.

- We have added the subtitle below.
- “Identification of the single residues of GABARAPL1 required for vIRF-1 binding”

8. Fig 5F. This is an important experiment, but its interpretation is confusing. It was described in the Results section only briefly. I note that HA-GABARAPL1 expression in the complemented KO cells is much more than the expression of endogenous GABARAPL1 in WT HeLa cells. Does this affect mitophagy and interpretation of the results? The authors claim that mitophagy was significantly inhibited in the KO cell line but was rescued by WT GABARAPL1 expression. I do not see it if I compare lane 5 with lane 1 and lane 6 with lane 5. There are some small differences, but I do not know if they are significant. I do not understand why the investigators show the significant tests for those they show. For example, comparing lane 11 with lane 2 basically show the effect of overexpressed HA-GABARAPL1 on mitophagy, which is the same as shown in lane 5 in Fig 5C. To my opinion the samples that would be really important to compare with each other are in lane 1 (WT cells, mock), 4 (WT cells with vIRF1/NIX), 5 (KO cells, mock), 20 (KO cells with vIRF1/NIX), and 21 (KO cells complemented with GABARAPL1 and vIRF1/NIX expressed). However, to make unambiguous conclusion**,** it would be important to have the same expression level of GABARAPL1 across different samples/cell lines. Alternatively, HA-GABAPARL1 should also be overexpressed in WT HeLa cells and use these cells for comparison.

- We appreciate this insightful comment. As suggested, we performed the experiments under the transfection condition that the expression levels of reconstituted GABARAPL1 in the KO cell lines were similar to the level of endogenous GABARAPL1 in control cells. Rather than mitophagy flux, we examined mitochondria content (as a cell population-based assay) by immunoblotting. As expected, we found that a decrease in mitochondria content induced by vIRF-1/NIX expression was inhibited in *GABARAPL1* KO HeLa.Kyoto cells but restored by reconstitution with sgRNA-refractory GABARAPL1 in a dose-dependent manner. (see new Fig 6E).

9. Does Fig 5C show one single blot (24-25-well protein gel?) or 3 blots together? If several blots were put together, I suggest using a line between the blots.

- We have added a line between the blots. See new Fig S5.

10. Fig 6C. vIRF1 runs at 75 kDa? It should be around 50 kDa as shown on other blots.

- We have fixed the protein marker labels. See new Fig 7C.

11. Fig 6D. If the NLS mutant of vIRF1 is not co-localized with mitochondria, how this vIRF1 mutants can induce more mitophagy? Does the NLS mutant interact (more/stronger) with GABARAPL1 and NIX?

- The vIRF-1 NLS^X^ variant could be detected in mitochondria by IFA (see new Fig 7D). We don’t know if the NLS^X^ speckles represent aggregated forms of vIRF-1 or if there is a technical issue in the IFA detection of the NLS^X^ speckles in mitochondria. To confirm the mitochondrial localization of the aggregated forms of the vIRF-1 NLS^X^ variant, we isolated mitochondria from transfected cells and performed immunoblotting. The result showed that the NLS^X^ could indeed be detected as aggregated forms in the enriched mitochondria fraction when co-transfected with NIX. See new Fig 7E.
- In addition, we performed NanoBiT assays to examine whether the NLS mutant interacts stronger with GABARAPL1 than native vIRF-1. Indeed, the NLS^X^ could significantly bind to GABARAPL1 compared to native vIRF-1 when co-transfected with NIX. See new Fig 7F.

12. Fig 7B. vIRF1 protein seems to be extremely stable that even 8 hours of CHX treatment did not change its protein level. If this is indeed the case, lane 339 “modified and aggregated vIRF1 exhibit a high protein turnover” is wrong. It should read low protein turnover.

- Sorry for the confusion. The vIRF-1 protein that seems to be highly stable is unmodified vIRF-1 (50 kDa). We changed the sentence to clarify this: “These results suggest that modified and aggregated vIRF-1 exhibit a higher protein turnover than unmodified vIRF-1.” Note that Fig 7B moved to Fig 9B.

13. How does the mutation in NLS or GABARAPL1 binding domain of vIRF1 affect the formation of aggregated vIRF1? How is this protein aggregation that does not seems to be associated with mitochondria connected to mitophagy?

- As mentioned above, immunoblotting analysis shows that aggregated forms of the vIRF-1 NLS^X^ variant could be highly detected in the mitochondria isolated from NIX-overexpressing cells compared to those of native vIRF-1. This might be simply due to a higher concentration of the NLS^X^ variant at the cytoplasm than native vIRF-1. In supporting this notion, aggregated forms of native vIRF-1 in virus-infected cells were readily detected when highly expressed 2 and 3 days after reactivation (Fig 8A). Nonetheless, we don’t exclude the possibility that the mutation at the NLS sequence (RGRRR) of vIRF-1 makes it prone to aggregate in the presence of NIX.
- We have mentioned the above in the Discussion section.
